# Race-Related Differences in Sipuleucel-T Response among Men with Metastatic Castrate–Resistant Prostate Cancer

**DOI:** 10.1158/2767-9764.CRC-24-0112

**Published:** 2024-06-10

**Authors:** Elisabeth I. Heath, Archana Thakur, Wei Chen, Clara Hwang, Channing J. Paller, Frank C. Cackowski, Julie L. Boerner, Lance Heilbrun, Melanie P. Smith, Dana L. Schalk, Amy Schienschang, Sarah A. Whitaker, Amanda Polend, Daryn Smith, Ulka N. Vaishampayan, Brenda Dickow, Lawrence G. Lum

**Affiliations:** 1 Department of Oncology, Karmanos Cancer Institute, Wayne State University School of Medicine, Detroit, Michigan.; 2 Department of Medicine, University of Virginia Cancer Center, Charlottesville, Virginia.; 3 Henry Ford Hospital, Detroit, Michigan.; 4 Johns Hopkins Sidney Kimmel Comprehensive Cancer Center, Baltimore, Maryland.; 5 Department of Medicine, University of Michigan School of Medicine, Ann Arbor, Michigan.

## Abstract

Sipuleucel-T is an autologous cellular immunotherapy that targets prostatic acid phosphatase (PAP) and is available for treatment of men with asymptomatic or minimally symptomatic metastatic castration-resistant prostate cancer (mCRPC). In this single-arm, two-cohort, multicenter clinical study, potential racial differences in immune responses to sipuleucel-T in men with mCRPC were explored. Patients’ blood samples were obtained to assess serum cytokines, humoral responses, and cellular immunity markers before and after treatment. Baseline cumulative product parameters (total nucleated and CD54^+^ cell counts and CD54 upregulation) were evaluated. IgM titers against the immunogen PA2024, the target antigen PAP, prostate-specific membrane antigen (PSMA) and prostate-specific antigen (PSA) were quantified by ELISA. Cytotoxic T-lymphocyte activity was determined by ELISpots, and cytokine and chemokine concentrations were determined by Luminex.Twenty-nine African American (AA) men and 28 non-African American (non-AA) men with mCRPC received sipuleucel-T. Baseline total nucleated cell count, CD54^+^ cell count, CD54 expression, and cumulative product parameters were higher in non-AA men. Although PSA baseline levels were higher in AA men, there were no racial differences in IgM antibody and IFNγ ELISpots responses against PA2024, PAP, PSA, and PSMA before and after treatment. Expression of co-stimulatory receptor ICOS on CD4^+^ and CD8^+^ T cells, and the levels of Th_1_ cytokine granulocyte-macrophage colony-stimulating factor and chemokines CCL4 and CCL5, were significantly higher in AA men before and/or after treatment. Despite no difference in the overall survival, PSA changes from baseline were significantly different between the two races. The data suggest that immune correlates in blood differ in AA and non-AA men with mCRPC pre- and post-sipuleucel-T.

**Significance:**

Our novel findings of higher expression of co-stimulatory receptor ICOS on CD4^+^ and CD8^+^ T cells in African American patients with metastatic castrate-resistant prostate cancer (mCRPC) prior and post-sipuleucel-T suggest activation of CD4^+^ and CD8^+^ T cells. The data indicate that racial differences observed in these and other immune correlates before and after sipuleucel-T warrant additional investigation to further our understanding of the immune system in African American men and other men with mCRPC.

## Introduction

Sipuleucel-T (Provenge^®^, Dendreon) is an autologous dendritic cell based cellular immunotherapy approved by the US Food and Drug Administration (FDA) in 2010 as the first-in-class treatment for patients with asymptomatic or minimally symptomatic metastatic castrate resistant prostate cancer (mCRPC; refs. 1, 2). The cell product is prepared by separation of a patient's mononuclear cells in a process called leukapheresis. These immune cells are then activated *ex vivo* by PA2024, a recombinant fusion protein that consists of prostatic acid phosphatase (PAP) fused to granulocyte-macrophage colony-stimulating factor (GM-CSF). After this exposure, the activated immune cells are infused into the patient to treat the prostate cancer (3). The therapeutic product is designed to activate antigen-presenting cells (APC) and subsequently produce a T-cell response against prostate cancer cells expressing PAP (4).

In the registration trial, sipuleucel-T improved median overall survival (OS) by 4.1 months in patients with asymptomatic or minimally symptomatic mCRPC but curiously did not reliably induce radiographic or prostate-specific antigen (PSA) responses (5). Sipuleucel-T was granted FDA approval because it provided a survival advantage to men with mCRPC and was subsequently recommended as a category 1 treatment option by the National Comprehensive Cancer Network Clinical Practice Guidelines in Oncology for Prostate Cancer (6). To better understand this novel first-in-class therapeutic, the PROVENGE Registry for the Observation, Collection, and Evaluation of Experience Data (PROCEED; NCT01306890) was formed after sipuleucel-T approval. The registry enrolled 1,976 patients with mCRPC of whom 1,902 received ≥1 sipuleucel-T infusion(s) and were followed for a median of 46.6 months. Sartor and colleagues utilized the PROCEED registry to compare OS in African American (AA) men (*n* = 219) and non-African American (non-AA) men (*n* = 438) treated with sipuleucel-T and found estimated OS medians of 35.3 months [95% confidence interval (CI), 28.7–42.7 months] and 25.8 months (95% CI, 22.6–29.0 months), respectively, in PSA-matched patients (7). These findings are especially intriguing because AA men classically have higher morbidity and mortality rates as compared to non-AA men but have similar outcomes from various treatment options (8). Furthermore, AA men with prostate cancer appear to have more active immune responses separate from sipuleucel-T or any other treatment, as reviewed by Sentana-Lledo and colleagues (9). In addition, Hawley and colleagues recently reported that, compared to non-AA patients (*n* = 36), AA patients (*n* = 18) treated with sipuleucel-T had higher serum concentrations of T_H_2-type cytokines (IL4, IL10, and IL13) and inflammatory cytokines (IL2, IL6, and IL12; ref. 10). To add to the limited immune marker data available for AA men with mCRPC, our study aimed to contribute additional information to better understand the effect of sipuleucel-T on the immune system.

## Materials and Methods

### Study design

Under the Wayne State University Institutional Review Board approval (IRB # 2013-108), a single arm, two-cohort, multicenter clinical study was conducted in AA and non-AA men with mCRPC treated with sipuleucel-T. The institutional review boards at all participating institutions approved the study. Participating institutions, which served as patient-recruiting sites, were Karmanos Cancer Institute, Henry Ford Cancer Institute, and Sidney Kimmel Comprehensive Cancer Center at Johns Hopkins University. Written informed consent was obtained by all patients prior to study procedures. The study was conducted in compliance with the Declaration of Helsinki, the International Conference on Harmonisation E6 Guideline for Good Clinical Practice, and the FDA Guidance for Industry: Good Pharmacovigilance Practices and Pharmacoepidemiologic Assessment. The primary objective was to determine the levels of immune parameters from sipuleucel-T-treated AA and non-AA patients with mCRPC. Secondary objectives were to compare the immune responses between the two race cohorts and the potential impact of race on OS. As this was a biomarker study of blood samples from patients undergoing an FDA-approved treatment, safety data were not collected.

### Patient selection

Eligible patients were 18 years or older with histologically confirmed prostate cancer. Patients who were undergoing sipuleucel-T treatment for mCRPC, as deemed by the treating investigator, were eligible to enroll. Evaluable patients included in the study were defined as those who had at least one post-baseline immune response sample collected. While on-study, patients were allowed to receive concurrent androgen receptor signaling inhibitors (ARSI) therapy in conjunction with sipuleucel-T [cf. (11)].

### Treatment plan

**Figure 1:**
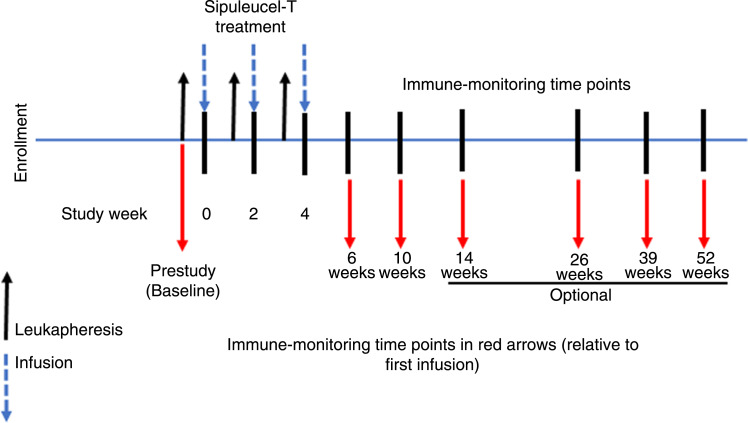
Study schema. Treatment schema showing schedule of sipuleucel-T infusions and immune evaluations. *Wk*, week.

Each dose of sipuleucel-T contained a minimum of 50 × 10^6^ autologous CD54^+^ cells activated with PAP-GM-CSF and was prepared by a single leukapheresis procedure. Sipuleucel-T was also composed of T cells, B cells, and natural killer cells that were harvested from each patient’s leukapheresis. A complete course of sipuleucel-T therapy consisted of three freshly prepared doses of sipuleucel-T administered via intravenous infusion at approximately 2-week intervals (Fig. 1). Blood sample collection was performed before treatment (at any time point prior to leukapheresis for the first sipuleucel-T dose) and at weeks 6, 10, 26, 39, and 52 post-treatment (after the first infusion). An 80-mL blood sample was collected from each patient at indicated time points. When available, we evaluated leukapheresis and cumulative product parameters, i.e., the sum of the values across the three sipuleucel-T doses for total nucleated cell (TNC) count (1 × 10^6^ cells/mm^3^), CD54^+^ cell count corresponding to APC number (1 × 10^6^ cells/mm^3^), and CD54 upregulation, which is equivalent to the APC activation value. When available, the baseline product components of sipuleucel-T were analyzed and expressed as medians. The APC activation value was measured as the increase in the surface CD54 on APCs. The value is equivalent to upregulation of CD54 and was expressed as the ratio of the average number of CD54 molecules on post-culture versus preculture cells.

### Serum antibody levels to prostate cancer antigens

We analyzed patient sera at baseline and after sipuleucel-T infusions against four antigens, i.e., PAP, PA2024, prostate-specific membrane antigen (PSMA), and PSA, along with positive and negative controls. Serially diluted sera (1:100–1:10,00,000) were screened for the presence of anti-PA2024, -PAP, -PSMA, and -PSA antibodies by standard ELISA as described previously (4). Serum samples were analyzed for endpoint titers of anti-PA2024, anti-PAP, anti-PSMA, and anti-PSA IgM antibodies, and data are shown as antibody titer of serum sample at optical density 405 nm.

### IFNγ ELISpots

The IFNγ ELISpots were used as surrogate markers for both CD8^+^-mediated memory cytotoxic T lymphocytes (CTL) activity and CD4^+^-mediated helper responses. Spontaneous, PA2024-, PAP-, PSMA-, and PSA-stimulated IFNγ ELISpots produced by peripheral blood mononuclear cells (PBMC) were assessed to serve as baseline controls. These ELISpot responses were also determined at the designated time points post-treatment and compared with their proper baseline levels. The cryopreserved T cells from patients were thawed and rested overnight, before being placed in the ELISpot plates at 3 × 10^5^ cells/well. PBMC (3 × 10^5^ cells/well) were stimulated with specific antigens (5–10 μg/mL) for 48 hours at 37°C and processed as described previously (12). Specific IFNγ-producing cells were assessed as the mean number of spots (triplicate wells) in the presence of antigen minus the mean number of spots in the absence of antigen per 3 × 10^5^ PBMC. Phytohemagglutinin-L and an irrelevant peptide were used as positive and negative controls, respectively, and cells without peptide were used as a background control at each time point. ELISpots were scored as positive if the ELISpots/million PBMC plated were ≥5 spots, and a positive increase was defined as a ≥2-fold increase in the IFNγ ELISpots over the baseline.

### T-cell phenotyping

PBMC from the patients treated with sipuleucel-T were sequentially evaluated to assess changes in phenotype. Antibodies used for staining included anti-CD45, -CD3, -CD4, -CD8, -CD278 (ICOS, inducible T-cell co-stimulator), -OX40 (CD134), -PD1 (CD279), -CTLA-4 (CD152), -CD272 (BTLA, B- and T-lymphocyte attenuator), -CD14, -HLA-DR and -CD54 (BioLegend). Cells were analyzed on a NovoCyte flow cytometer (Agilent Technologies), and data were analyzed using NovoExpress software (Agilent Technologies). Co-stimulatory receptor expression by T cells was determined by analyzing ICOS or OX40 surface expression after gating on CD45^+^/CD3^+^/CD4^+^ or CD45^+^/CD3^+^/CD8^+^ T cells; and co-inhibitory receptor expression by analyzing PD-1, CTLA-4, or BTLA after gating on CD45^+^/CD3^+^/CD4^+^ or CD45^+^/CD3^+^/CD8^+^ T cells. Changes in the proportion of CD4^+^ and CD8^+^ T cells expressing co-stimulatory or co-inhibitory receptors were analyzed by multicolor flow cytometry at the indicated time points. The activation of APCs was analyzed by gating on CD45^+^/HLA-DR^+^/CD54^+^ cells.

### Serum cytokine profiles

Cytokines were measured in serum samples at selected time points using a 25-plex human cytokine Luminex assay (R&D) and the BioPlex system (Bio-Rad). The multiplex panel included interleukin (IL)-1 receptor antagonist (IL1RA), IL2, IL4, IL6, IL7, IL8 (CXCL8), IL13, IL17, tumor necrosis factor-alpha (TNFα), interferon-alpha (IFNα), IFNγ, GM-CSF, macrophage inflammatory protein-1 alpha (MIP-1α, CCL3), MIP-1β (CCL4), interferon gamma-induced protein 10 kDa (IP-10, CXCL10), eotaxin (CCL11), regulated on activation normal T cell expressed and secreted (RANTES, CCL5), and monocyte chemoattractant protein-1 (MCP-1, CCL2). The cytokine levels were interpolated from a standard curve using BioPlex Manager Software (Bio-Rad).

### Statistical methods

A single arm, single-stage, two-cohort design was planned. The primary objective was to describe the immune parameters in AA and non-AA patients with mCRPC separately at each time point. Numerical and graphical descriptive statistics were generated for baseline characteristics and immune parameters at each time point. Immune biomarkers were compared between racial groups using Wilcoxon rank sum test at each time point. Changes of immunological markers at 10 weeks post infusion versus baseline levels were analyzed using Wilcoxon signed rank test within each race group. A linear mixed model was used to evaluate the difference of longitudinal PSA values between the race groups. Spearman coefficient was used to evaluate the correlation between the baseline PSA and an immunological marker at different time points. OS, defined as the time from study enrollment to death or censored at the last follow up, was assessed using the Kaplan–Meier method. The median OS and its 95% CI in each cohort was reported. Due to nonproportional hazards of race, a time-dependent Cox regression model was performed to estimate the hazard ratio (HR) of AA to non-AA men, where race was modeled to have a time dependent effect. The Cox model was adjusted for baseline PSA as a fixed covariate. All statistical tests were exploratory in nature and hypothesis-generating. We expected approximately 90 comparisons for the proposed immunological markers at each time point. Based on the marginal false discovery rate (mFDR) method (13), an alpha level of 0.0032 with 80% power for each test would result in a 5% mFDR assuming five true alternative hypotheses. *P* values <0.0032 were considered statistically significant for the Wilcoxon rank sum tests and the Wilcoxon signed rank tests, unless otherwise noted.

### Data availability

The data generated in this study are available upon request from the corresponding author.

## Results

### Clinical characteristics

Between November 4, 2013, and October 2, 2020, 57 patients (29 AA men and 28 non-AA men) received ≥1 sipuleucel-T infusion at the three recruiting sites. Patient demographics, pathology variables, and prior treatments are shown in Table 1. The median age of 68 years was the same in both groups. Compared to non-AA men, AA men had higher median baseline PSA level (20.5 ng/mL vs. 8.0 ng/mL) and more AA men had lymph node involvement (38% vs. 29%). For prior treatment of mCRPC, AA patients received docetaxel more often than their non-AA counterparts (17% vs. 4%), whereas more non-AA men were treated with abiraterone than AA men (18% vs. 10%). Prior enzalutamide and prior bicalutamide were each received by a small percentage of patients in both groups (7% AA and 4% non-AA, respectively). Three patients had ongoing treatment with androgen receptor signaling inhibitors (ARSI) during the study. One patient received enzalutamide and two patients received abiraterone. There were no appreciable differences in baseline total white blood cell count or hemoglobin values. Because patients’ baseline characteristics shown in Table 1 are descriptive and not intended for hypothesis-testing by comparison of the two groups, no *P*-values are shown. As the sample size was small, *P* values tend to be large and unable to reveal potential differences and are generally not recommended (cf.; refs. 14, 15).

**Table 1:** Baseline clinicopathologic characteristics of patients with mCRPC and their sipuleucel-T cumulative product parameters

Characteristics	African Americans (*N* = 29)	Non-African Americans (*N* = 28)	Total (*N* = 57)
Median age–years (range)	68 (48–87)	68 (54–83)	68 (48–87)
Median baseline PSA–ng/mL (range)	20.5 (0.7–4,915)	8.0 (0.5–191)	14.3 (0.5–4,915)
Gleason grade–*n* (%)			
≤6	3 (12)^a^	0 (0)	3 (6)
7	4 (15)	9 (32)	13 (24)
8–10	19 (73)	19 (68)	38 (70)
Metastatic sites–*n* (%)			
Bone	22 (76)	23 (82)	45 (79)
Visceral	1 (3)	2 (7)	3 (5)
Lymph nodes	11 (38)	8 (29)	19 (33)
Prior treatments–*n* (%)			
Enzalutamide	2 (7)	1 (4)	3 (5)
Abiraterone	3 (10)	5 (18)	8 (14)
Docetaxel	5 (17)	1 (4)	6 (11)
Bicalutamide	2 (7)	1 (4)	3 (5)
Median white blood cell count (1,000/mm^3^)			
Neutrophils	3.6	3.8	3.6
Lymphocytes	1.6	1.4	1.5
Eosinophils	5.9	5.4	5.5
Median baseline hemoglobin (g/dL)	11.8	13.1	12.0
Cumulative sipuleucel-T product parameters^b^			
TNC (1×10^6^ cells/mm^3^)	11,974	12,774	
APC (1×10^6^ cells/mm^3^)	1.973	2,116	
APC (activation value)	32.0	37.5	

a Gleason scores for three African Americans were missing, and therefore the percentage was calculated for 26 not for 29 African Americans.

b In 19 African Americans and 21 non-African Americans.

### Cumulative product parameters of sipuleucel-T

To measure the product parameters of sipuleucel-T, we determined the activated APC in the context of the TNC and the total APC. In the AA group (*n* = 19), the median absolute value of TNC was 11,974 (1 × 10^6^ cells/mm^3^) compared to 12,774 (1 × 10^6^ cells/mm^3^) in the non-AA group (*n* = 21) (Table 1). The median of the cumulative APC value was lower in AA patients compared to non-AA patients (1,973 × 10^6^ cells/mm^3^ vs. 2,116 × 10^6^ cells/mm^3^). Similarly, the median of the cumulative APC activation value was lower in AA men compared to non-AA men (32.0 vs. 37.5). Thus, the medians for APC value and activation were 7% and 15% lower, respectively, in AA vs. non-AA patients.

### Antibody responses increase after sipuleucel-T infusions

**Figure 2:**
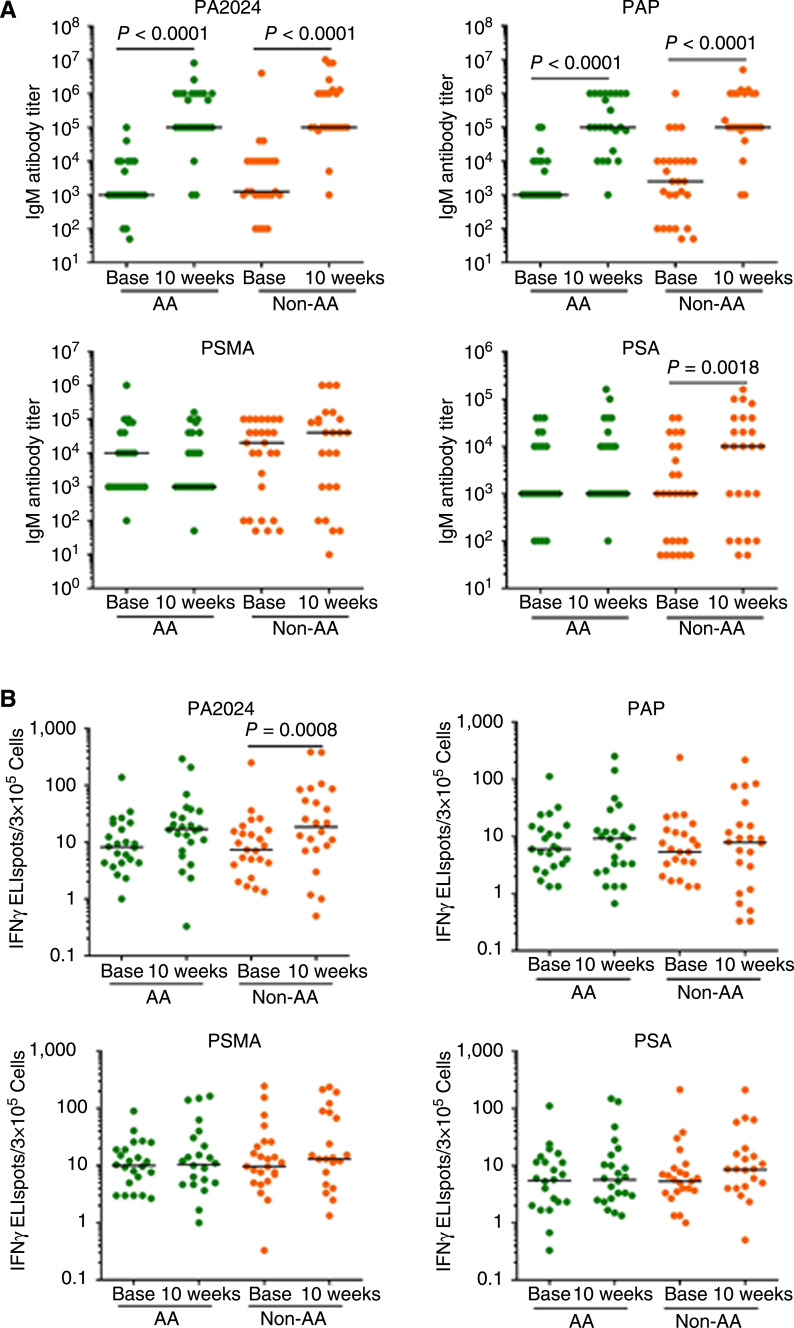
Peripheral antibody and IFNγ ELISpot immune responses in AA men (*n* = 29) and non-AA men (*n* = 28). **A,** Significantly higher serum IgM antibody levels against PA2024 (*P* < 0.0001) and PAP (PAP; *P* < 0.0001) were noted at 10 weeks post-treatment above the baseline in each group. No significant differences were observed in either group for anti-PSMA antibody levels at 10 weeks post-treatment vs. baseline. There were significantly higher anti-PSA antibody levels in non-AA group (*P* = 0.0018) at 10 weeks post-treatment above the baseline. **B,** IFNγ ELISpot responses to PA2024 increased significantly (*P* = 0.0008) in non-AA men at 10 weeks post-treatment vs. baseline. In AA men, IFNγ ELISpot responses to PA2024 also increased (*P* = 0.04) at 10 weeks post-treatment. No significant differences in IFNγ ELISpot responses were seen at 10 weeks against PAP, PSMA, or PSA in either group. For (**A** and **B**), there were no significant racial differences in either antibody or IFNγ ELISpot responses against PA2024, PAP, PSMA, or PSA at baseline or at 10 weeks post-treatment. All responses and racial differences were analyzed by the Mann–Whitney test and *P* values < 0.0032 were considered as significant in the multiple comparisons sense. The horizontal line in each dot plot marks the median value. *Base*, baseline; *wks*, weeks.

Serum IgM antibody levels against the sipuleucel-T specific epitope PA2024 were significantly higher (*P* < 0.0001) at 10 weeks post-treatment in both AA and non-AA patients. At baseline, the median antibody titer was approximately 1 × 10^3^ in each group, whereas at 10 weeks post-treatment, the median antibody titer was 1 × 10^5^ and 1 × 10^6^ in AA and non-AA patients, respectively. Anti-PAP IgM response also increased significantly (*P* < 0.0001) at 10 weeks post-treatment in both groups, with median antibody titer of 1 × 10^5^ in each group. No significant changes in anti-PSMA antibody levels were seen post sipuleucel-T infusion in either group. Only in the non-AA group, anti-PSA antibody levels were significantly higher (*P* = 0.0018) at 10 weeks post-treatment. No differences were observed between the two groups before or after treatment in IgM antibody levels against PA2024, PAP, PSA, or PSMA (Fig. 2A; Supplementary Table S1).

### CTL activity by IFNγ ELISpot responses was higher in non-AA patients after sipuleucel-T infusions

CTL responses were measured by IFNγ ELISpot assays. CTL activity in response to PA2024 increased significantly at 10 weeks post-treatment (*P* = 0.0008) in non-AA patients. AA patients also showed increased CTL activity in response to PA2024 (*P* = 0.04) post-treatment but the increases were not considered significant. No significant changes were observed in CTL activity in responses against PAP, PSMA, or PSA in either group. Overall, the activity of CD8^+^ T cells as measured by IFNγ ELISpot was similar in the two racial groups after sipuleucel-T infusion (Fig. 2B; Supplementary Table S1).

### Differential patterns of CD8^+^ T-cell proportions and of expression of CD4^+^ and CD8^+^ T-cell co-stimulatory receptors

**Figure 3:**
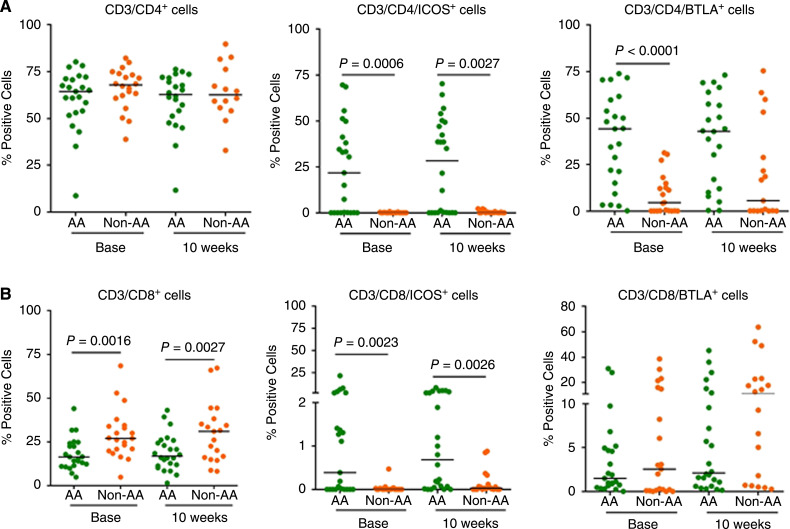
Co-stimulatory and co-inhibitory marker expression on CD4^+^ and CD8^+^ T cells in two racial groups. **A,** No differences in the percentage of CD4^+^ T cells were observed at baseline or at 10 weeks post-treatment in AA men (*n *= 29) vs. non-AA men (*n* = 28). Expression of co-stimulatory receptor ICOS on CD4^+^ T cells was significantly higher at baseline and at 10 weeks post-treatment (*P* = 0.0006; *P* = 0.0027, respectively) in AA men compared to non-AA men. Expression of co-inhibitory receptor BTLA on CD4^+^ was also significantly higher at baseline (*P* < 0.0001) in AA men compared to non-AA men. **B,** The percentage of CD8^+^ T cells was significantly lower (*P* = 0.0016) at baseline as well as at 10 weeks post- treatment in AA men compared to non-AA men. Expression of co-stimulatory receptor ICOS on CD8^+^ T cells was significantly higher at baseline and at 10 weeks post-treatment (*P* = 0.0023; *P* = 0.0026, respectively) in AA men vs. non-AA men. Differences between the two racial groups were analyzed by the Mann–Whitney test and *P* values < 0.0032 were considered as significant in the multiple comparisons sense. The horizontal line in each dot plot marks the median value. *Base*, baseline; *wks*, weeks.

At baseline, while there was no difference in the percentage of CD4^+^ T cells, the percentage of CD8^+^ T cells was significantly higher at baseline and at 10 weeks post-treatment (*P* = 0.0016 and *P* = 0.0027, respectively) in non-AA than AA patients. To evaluate activation of CD4^+^ and CD8^+^ T cells, the expression of co-stimulatory receptor ICOS on CD4^+^ and CD8^+^ T cells before and after treatment was assessed. The percentage of CD4^+^ T cells expressing ICOS was significantly higher in AA patients than non-AA patients at baseline (*P* = 0.0006) and 10 weeks (*P* = 0.0027). Similarly, the percentage of CD8^+^ T cells expressing ICOS was significantly higher at baseline (*P* = 0.0023) and 10 weeks (*P* = 0.0026) in AA patients than non-AA patients. Of note, in the non-AA group the expression of ICOS on CD4^+^ and CD8^+^ cells was low at baseline and did not change after treatment (Fig. 3A and B; Supplementary Table S1). Although not significant, a numerically higher percentage of co-stimulatory receptor OX40-expressing CD4^+^ and CD8^+^ T cells was observed before and after treatment in the AA group versus non-AA group (Supplementary Fig. S1). Taken together, the data show that the percentage of co-stimulatory receptor-expressing CD4^+^ and CD8^+^ T cells was higher in AA patients than in non-AA patients before and after sipuleucel-T.

### Differential expression patterns of co-inhibitory receptors on CD4^+^ and CD8^+^ T cells

BTLA, a co-inhibitory receptor that signals CD4^+^ and CD8^+^ inactivation, was expressed at a significantly higher number (*P* < 0.0001) on CD4^+^ T cells at baseline and remained high after treatment in AA patients. BTLA expression was not elevated, however, on CD8^+^ T cells before or after treatment in either group (Fig. 3A and B; Supplementary Table S1). In contrast, expression of PD1 and/or CTLA-4 co-inhibitory receptors on CD4^+^ and CD8^+^ T cells were numerically higher in non-AA patients before and/or after treatment but the difference was not significant between the two groups (Supplementary Fig. S1).

### Differential cytokine and chemokine responses

A panel of multiple cytokine and chemokine biomarkers were quantitated in serum using the Luminex system. Th1 and Th2 cytokines that are most likely to participate in immune responses are presented. The levels of Th1 cytokines (IL2, IL12, GM-CSF, and TNFα), cytokines with antitumor activity (IL1RA, IL15, and IFNα), Th2 cytokines (IL6 and IL10), in addition to chemokines (IP-10, MIP-1α, CCL4, CCL5, and IL8) were all analyzed at baseline and 10 weeks post-infusion.

**Figure 4:**
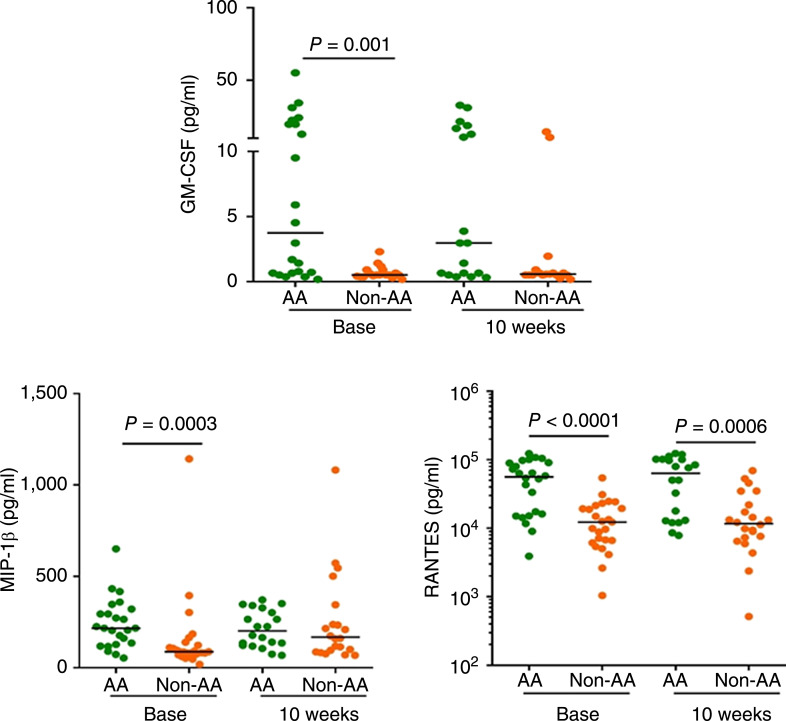
Differential cytokine and chemokine responses in AA men (*n* = 29) and non-AA men (*n* = 28). Of all Th_1_ cytokines, only the baseline levels of GM-CSF were significantly higher (*P* = 0.001) in AA men vs. non-AA men. The levels of chemokine CCL4 (MIP-1β) were significantly higher at baseline (*P* = 0.0003) in AA men vs. non-AA men. The levels of CCL5 (RANTES) were significantly higher at baseline (*P* < 0.0001), as well as at 10 weeks post treatment (*P* = 0.0006) in AA men vs. non-AA men. Differences between the two racial groups were analyzed by the Mann–Whitney test and *P* values < 0.0032 were considered as significant in the multiple comparisons sense. The horizontal line in each dot plot marks the median value. *Base*, baseline; *wks*, weeks.

GM-CSF was the only Th_1_ cytokine that was significantly higher (*P* = 0.001) at baseline in AA patients than in non-AA patients and remained elevated, albeit not significantly, at 10 weeks. The levels of T-cell recruiting chemokine CCL4 (MIP-1β) were significantly higher (*P* = 0.0003) at baseline in AA patients than in non-AA patients. Furthermore, the levels of CCL5 (RANTES) in AA patients were significantly elevated at baseline (*P* < 0.0001) and at 10 weeks (*P* = 0.0006) post treatment compared to non-AA patients (Fig. 4; Supplementary Table S2).

Similar trends were found in the expression of CCL3 (MIP-1α), IP-10, and IL8 in the AA group relative to the non-AA group although not statistically significant. Numerically higher levels of IL2, IL12, and TNFα were also noted at baseline in the AA group. In the case of Th2 cytokines, the AA patients exhibited numerically higher levels of IL6 and IL10 at baseline than non-AA patients. We observed numerically higher IL1RA levels at baseline in AA patients than non-AA patients, and numerically higher IFNα and IL15 levels in the non-AA patients than AA patients at baseline and 10 weeks (Supplementary Fig. S2; Supplementary Table S2). IL1RA, IFNα, and IL15 regulate innate and adaptive immune responses and exhibit antitumor activity, including direct tumor cell killing and stimulating immune cells, e.g., dendritic cells and CD8^+^ T cells (16–21).

### PSA response to sipuleucel-T

**Figure 5:**
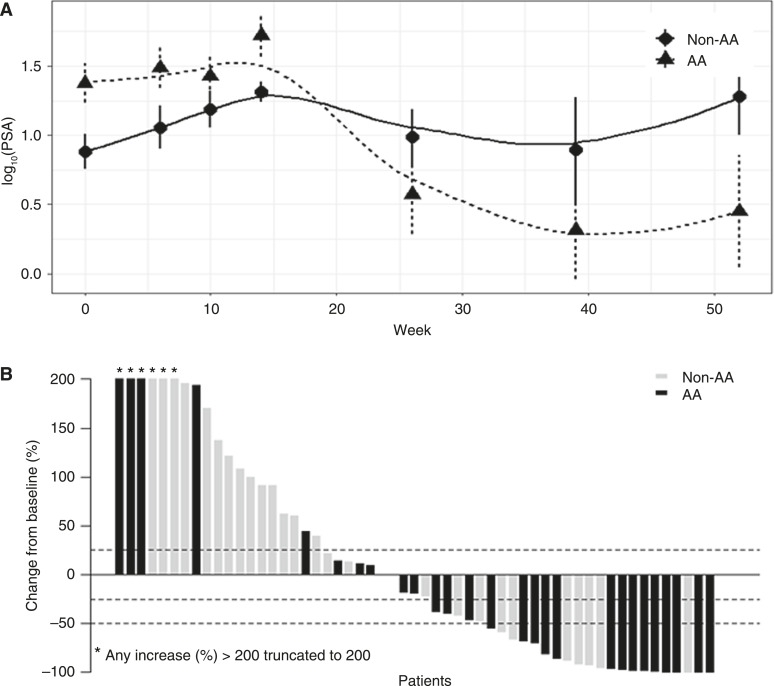
**A,** Longitudinal PSA levels by race groups. Curves were fitted with LOESS smoothing method. Dot and triangle symbols indicate mean log_10_(PSA) at the given time point. Vertical error bars indicate standard errors. **B,** Waterfall plot: PSA change from baseline to nadir.

As a biomarker for prostate cancer progression, PSA is a standard measurement when following prostate cancer patients. Longitudinal PSA levels by race were measured through the course of the study. Linear mixed model fit by restricted maximum likelihood (REML) resulted in significant difference between the racial groups. The PSA level increased weekly by an average of 1.2% for non-AA patients, whereas it decreased by 4.9% for AA patients. Interaction between the time and race was significant (*P* value = 0.011 using Satterthwaite method, Fig. 5A). The waterfall plot for PSA changes from baseline to nadir shows that more AA patients achieved PSA nadir of >50% decrease Fig. 5B. Prior to entry or on-study ARSI apparently did not affect the PSA response. The PSA percentage change from baseline to nadir reports the best outcome, regardless of which week the nadir was achieved. To provide additional clarity, we added waterfall plots for the PSA percentage change from baseline to nadir and for the PSA percentage change from baseline to week 10 (Supplementary Figs. S3A and S3B).

There was no significant correlation between marker changes from baseline to week 10 and baseline PSA (|rho|≤0.35; Table 2). Although there were several marker changes that moderately correlated with PSA fold change (week 10 to baseline), we used marker differences (from baseline to week 10) rather than marker fold changes (week 10/baseline) for two reasons. First, to be comparable to the Wilcoxon’s signed rank tests reported in this manuscript. Second, to accommodate the 0% positive cells for some markers, such as CD45CD3CD4ICOS and CD45CD3CD8ICOS, as zeros cannot be used as denominators in the fold change without adjustments.

**Table 2:** Spearman correlation between marker difference (diff; week 10-baseline) and PSA baseline or PSA fold change (PSA.FC; week 10/baseline; rho, *P* value).

	Marker diff vs. PSA baseline	Marker diff vs. PSA.FC
CD45CD3CD8	0.21, 0.24	−0.05, 0.787
CD45CD3CD4ICOS	0.25, 0.156	−0.09, 0.626
CD45CD3CD8ICOS	−0.05, 0.791	0.09, 0.634
CD45CD3CD4BTLA	0.02, 0.91	0.18, 0.327
IL.2	0.06, 0.737	−0.05, 0.798
IL.12p40p70	0.03, 0.888	0.46, 0.013
TNF-α	0.02, 0.932	0.47, 0.007
GM-CSF	−0.11, 0.536	0.23, 0.21
MIP-1β	−0.11, 0.488	0.39, 0.013
RANTES	−0.08, 0.617	−0.15, 0.357
PA2024 (ELISPOTS)	0.18, 0.268	−0.07, 0.681
PA2024 (ELISA)	0.02, 0.888	0.05, 0.764
PAP (ELISA)	0.07, 0.657	−0.05, 0.74
PSA (ELISA)	0.02, 0.887	0.17, 0.31

### OS after sipuleucel-T by race

The median OS of AA patients was 39.7 months (with 95% CI, 19.4–81.1 months; Supplementary Fig. S4). For non-AA patients, the median OS was 43.2 months (with 95% CI, 31.8–53.2 months). The time-dependent Cox regression model yielded an HR of 2.22, with 95% CI (0.89–5.54) for AA patients relative to non-AA patients, adjusting for baseline PSA levels. Hence, no difference in OS between AA patients and non-AA patients was observed in our study.

## Discussion

AA men have a higher prevalence of prostate cancer, greater likelihood of advanced prostate cancer at presentation, and greater propensity for progression and lethality compared with non-AA men (22, 23). In spite of that, AA men are underrepresented in clinical trials leading to a paucity of clinical and biological data for AA men with mCRPC (24). This study systematically examined the peripheral blood and serum for differences in immune phenotypes, cellular and humoral immune responses to sipuleucel-T in cohorts of AA and non-AA men with mCRPC. Our working hypothesis was that there would be differences in immune responses that may help explain differences in clinical responses between AA and non-AA patients. Overall, we observed higher serum concentrations of inflammatory cytokines and higher expression of co-stimulatory molecules on T cells of AA patients. In agreement with prior work, our results suggest improved response to sipuleucel-T in AA patients, though our study was not designed to show improved responses.

When biomarker levels and immune functions were compared to non-AA patients, AA patients had (i) significantly higher percentage of CD4^+^ and CD8^+^ of T cells expressing ICOS at baseline and at 10 weeks following sipuleucel-T therapy; (ii) significantly higher percentage of CD4^+^ T cells expressing BTLA at baseline; (iii) significantly increased levels of Th1 cytokine GM-CSF at baseline, and (iv) significantly increased levels of CCL4 at baseline, in addition to significantly increased levels of CCL5 at baseline and at 10 weeks after sipuleucel-T treatment (Supplementary Table S3).

Our novel finding of higher expression of co-stimulatory receptor ICOS on CD4^+^ and CD8^+^ T cells in AA patients before and after sipuleucel-T suggests increased baseline activation of CD4^+^ and CD8^+^ T cells. BTLA expression was also higher on CD4^+^ T cells in AA patients at baseline and 10 weeks after sipuleucel-T therapy. This finding suggests that increased BTLA expression might be a compensatory or attenuating response to ICOS-related heightened T-cell activation in AA patients. Binding of BTLA to its ligand herpes virus entry mediator inhibits T-cell proliferation and effector function (25, 26). The absence of racial differences before and after sipuleucel-T therapy, however, suggests that the attenuating response of BTLA expression was not due to treatment itself but rather an endogenous response inherent to AA patients. The results showing elevated levels of Th_1_ cytokine GM-CSF and chemokines CCL4 and CCL5 in the sera of AA patients at baseline and/or after sipuleucel-T treatment are consistent with the notion of prior or ongoing *in vivo* immune activation. CCL4 and CCL5 are involved in attracting endogenous T cells and monocytes to the tumor site, while GM-CSF mediates the differentiation and activation of APC, as well as activation of T cells (27).

The observation of increased levels of cytokines at baseline in AA patients is broadly consistent with a report from Hawley and colleagues (10). Using peripheral blood samples from 54 men with mCRPC enrolled in PROCEED registry, they observed elevated levels of Th2-type (IL4, IL10, and IL13) and inflammatory cytokines (IL2, IL6, and IL12) in AA patients compared to their non-AA counterparts at baseline and at 52 weeks following sipuleucel-T therapy. We did observe increased baseline and post sipuleucel-T treatment serum inflammatory cytokines in AA patients, though the cytokines were not the same (CCL4 and CCL5 rather than IL2, IL6, and IL12). We also did not observe statistically significant increases in Th2 cytokines but did observe an increased concentration of GM-CSF, which can function as a Th1 cytokine. We also observed high expression of co-stimulatory molecule ICOS on T cells from AA patients—a parameter not reported by Hawley and colleagues (10).

As this was a nonrandomized study, selection bias is one of its limitations. In addition, the observed responses of circulating immune biomarkers do not necessarily reflect the on-treatment tumor immune microenvironment. Tumor biopsies may reveal genetic underpinnings of potential racial differences in immune responses but that was beyond the scope of the study. Furthermore, more elaborate cytokine and/or proteomic analysis could uncover other biomarkers that are better associated with the improved outcome in AA men.

In contrast to our study, previous research has demonstrated a link between immune responses and OS. For instance, GuhaThakurta and colleagues (28) observed that serum IgG antibody responses to secondary tumor antigens, indicative of humoral antigen spread, can be detected within weeks after treatment with sipuleucel-T in mCRPC. These responses correlated with longer OS, suggesting their potential as biomarkers of clinical outcomes post-sipuleucel-T treatment. Similarly, Antonarakis and colleagues (29) reported persistent antigen-specific CD4^+^ T-cell responses and the emergence of antigen-specific CD8^+^ T cells with potential lytic capabilities following sipuleucel-T therapy. They identified a positive association between OS and both PAP- and PA2024-specific cytotoxic T-lymphocyte phenotypes, hinting at sipuleucel-T's capacity for tumor lysis. These findings propose a mechanism of action for sipuleucel-T that warrants further exploration.

The small sample size limited our ability to control for potential confounders. One of these is tumor burden, which can be approximated by serum PSA concentration. In our study, baseline PSA was much higher in AA. We also found that more AA patients achieved PSA nadir of >50% decrease after sipuleucel-T. Thus, greater number of AA patients contributed to high PSA response rate that we observed. Interestingly, greater percentage of AA men exhibited prolonged PSA stabilization that was associated with favorable overall prognosis post sipuleucel-T. These findings suggest that host factors associated with AA race could positively affect the clinical benefit of sipuleucel-T as was reported in a retrospective study by Holl and colleagues (30). Patient selection as a factor can also be considered.

Furthermore, due to the exploratory nature of the current analysis, the comparisons of the markers between the two cohorts were performed with univariate analysis only rather than adjusted for baseline PSA. There was no significant correlation, however, between the baseline PSA and immune markers (Table 2). Of note, in the PROCEED registry study that showed longer survival in AA men compared with non-AA men after sipuleucel-T (7), the patients were matched for PSA. In our study, one potential reason for no difference in OS between the two races could be elevated baseline PSA levels in AA cohort.

A randomized phase II trial in men with bone only mCRPC showed that treatment with radium-223 plus sipuleucel-T was associated with lower peripheral T-cell immune responses to the target antigen PA2024 compared with treatment with sipuleucel-T alone (31). Other T-cell biomarkers of immune response were similar in both treatment groups. In spite of the absence of enhanced peripheral and humoral responses in patients who received the combination, 33% of them had a PSA50 response when treated with sipuleucel-T itself. Furthermore, the combined treatment, unlike sipuleucel-T alone, led to significantly improved progression-free survival. Paradoxically, lower immune response was associated with lower PSA levels and better clinical outcome. Conversely, greater immune response was accompanied by higher PSA levels, and less-favorable clinical outcome. Based on these observations, apparently higher baseline PSA levels in AA patients might correlate with greater immune response, as was the case with baseline and post-treatment increased expression of co-stimulatory receptor ICOS on CD4^+^ and CD8^+^ T cells, in addition to elevated baseline and/or post-treatment levels of serum Th1 cytokine GM-CSF, as well as chemokines CCL4 and CCL5.

PSA-specific T cells induction following the metronomic cyclophosphamide has been observed in patients with biochemically recurrent prostate cancer (32). The treatment was associated with a decrease in regulatory T cells, resulting in reversal of immunosuppression, i.e., elevated levels of activated HLADR^+^ CD45RO^+^ T cells in peripheral blood, and the concomitant reactivation and rise in PSA-specific T cells, i.e., reactivation of Th1 polarized anti-PSA T cells. This specific T-cell activation led to lessening of PSA levels and control of disease progression. It was suggested that the combined effect of antiangiogenic and immune activation of this treatment has a potential of counteracting immune resistance associated with prostate cancer and can lead to improved clinical outcomes.

In conclusion, our data show that AA men with mCRPC have increased expression of BTLA on CD4^+^ T cells, increased levels of serum inflammatory cytokines CCL4 and CCL5, as well as of Th1 cytokine GM-CSF, in addition to increased expression of co-stimulatory molecule ICOS on CD4^+^ and CD8^+^ T cells after sipuleucel-T and/or at baseline. The data are broadly consistent with prior studies showing increased immune activity in AA men with prostate cancer, though precise molecular profiles differ. This dataset serves as a foundation for future work designed to increase efficacy of immune-based therapies in AA men and other men with prostate cancer.

## Supplementary Material

Supplementary Figure S1 Co-stimulatory and co-inhibitory marker expression on CD4+ and CD8+ T cells in AA (n=29) and non-AA (n=28).

Supplementary Figure S2 Cytokine and chemokine responses in AA (n=29) and non-AA(n=28).

Supplementary Figure S3A Waterfall plot showing PSA change from baseline to nadir.

Supplementary Figure S3B Waterfall plot showing PSA change from baseline to week 10.

Supplementary Figure S4 Overall survival post-sipuleucel-T by race.

Supplementary Table S1 Antibody and IFN-γ responses and co-stimulatory and co-inhibitory marker expressions in mCRPC patients.

Supplementary Table S2 Th1 and Th2 cytokine and chemokine responses in mCRPC patients.

Supplementary Table S3 Summary of significant responses before and after sipuleucel-T in mCRPC patients.
