# Supplementary material for: Race-Related Differences in Sipuleucel-T Response among Men with Metastatic Castrate–Resistant Prostate Cancer
Source: Cancer Res Commun. 2024 Jun 10;4(7):1715–25. doi: 10.1158/2767-9764.CRC-24-0112 (PMC11240276; doi:10.1158/2767-9764.CRC-24-0112)
Supplement: Supplementary Figure S1 — Co-stimulatory and co-inhibitory marker expression on CD4+ and CD8+ T cells in AA (n=29) and non-AA (n=28). [file crc-24-0112_supplementary_figure_s1_supps1.pdf]

Supplementary Figure S1

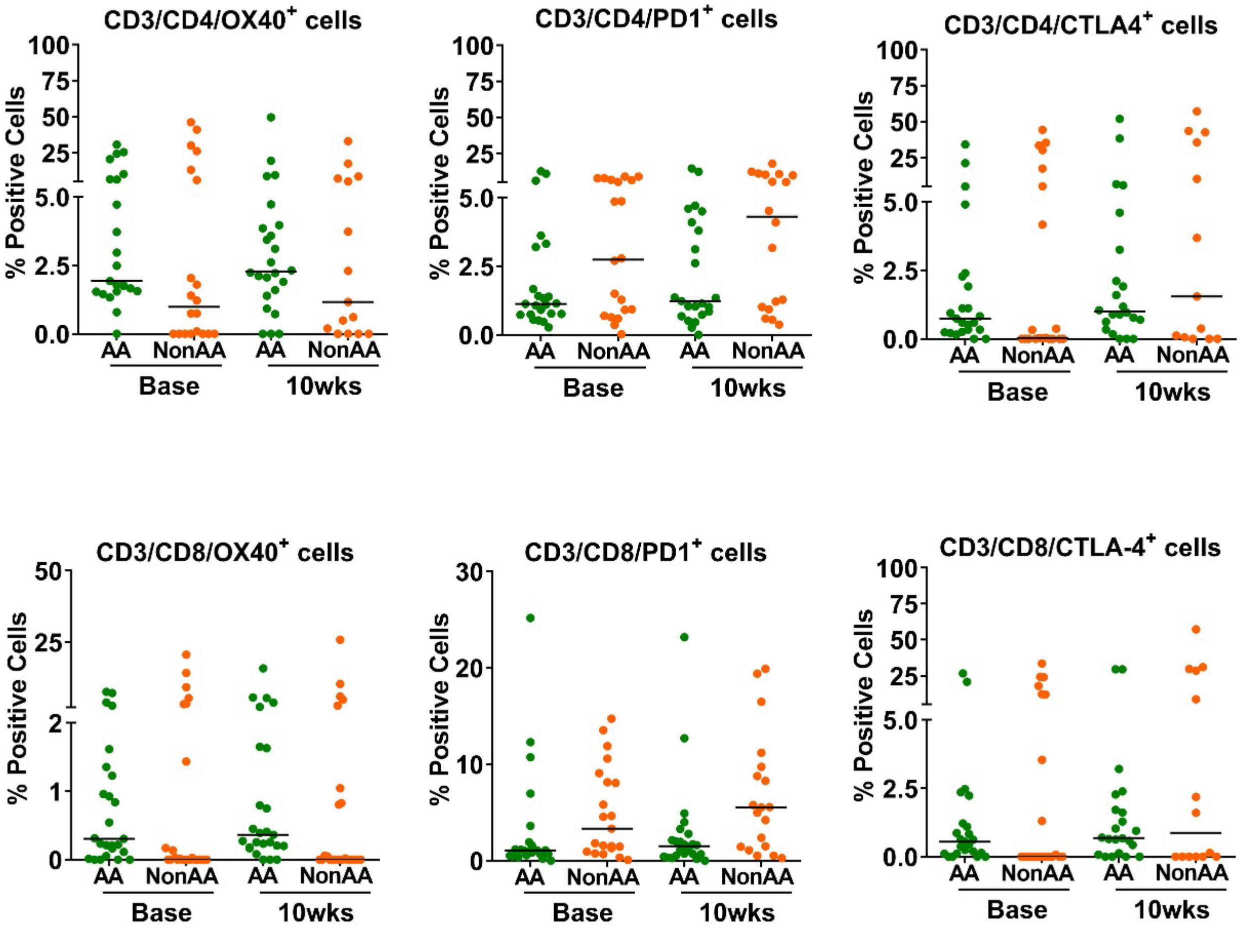

Supplementary Figure S1. Co-stimulatory and co-inhibitory marker expression on $CD4^+$ and $CD8^+$ T cells in AA (*n*=29) and non-AA (*n*=28). No statistical changes were observed for OX40 at baseline or 10 weeks post sipuleucel-T in AA patients vs non-AA patients in either CD4 or CD8 T cells. Expression of co-inhibitory receptors PD1 and CTLA-4 on $CD4^+$ or $CD8^+$ T cells showed no statistically significant changes between two groups. The OX40 expressing $CD4^+$ or $CD8^+$ T cells showed a trend of increased percentage in AA vs non-AA patients, while expression of PD-1 and CTLA4 co-inhibitory receptors on $CD4^+$ and $CD8^+$ T cells showed a trend of higher expression in non-AA compared to AA patients at baseline and/or 10 weeks post treatment. Differences between the two racial groups were analyzed by the Mann Whitney test and p values < 0.0032 were considered as significant in the multiple comparisons sense. The horizontal line in each dot plot marks the median value. Base, baseline; wks, weeks.

Supplementary Figure S2 (next slide). Cytokine and chemokine responses in AA (*n*=29) and non-AA (*n*=28). No significant racial differences were observed for the levels of chemokines MIP-1a, IP-10, and IL-8, Th_1 cytokines IL-2, IL-12 and TNF-a, Th_2 cytokines IL-6 and IL-10, or cytokines with anti-tumor activity-IL-1Ra, IFN-a and IL-15 at baseline or at 10 weeks post treatment. A trend of increased levels of IL-1Ra was noted in AA vs non-AA patients at baseline. In contrast, a trend of increased levels of IFN-a and IL-15 was observed in non-AA patients vs AA patients at baseline and 10 weeks post treatment. Differences between the two racial groups were analyzed by the Mann Whitney test and p values < 0.0032 were considered as significant in the multiple comparisons sense. The horizontal line in each dot plot marks the median value. Base baseline; wks weeks.
