# Supplementary figures and images for: Race-Related Differences in Sipuleucel-T Response among Men with Metastatic Castrate–Resistant Prostate Cancer
Source: Cancer Res Commun. 2024 Jun 10;4(7):1715–25. doi: 10.1158/2767-9764.CRC-24-0112 (PMC11240276; doi:10.1158/2767-9764.CRC-24-0112)

Supplementary Figure S2

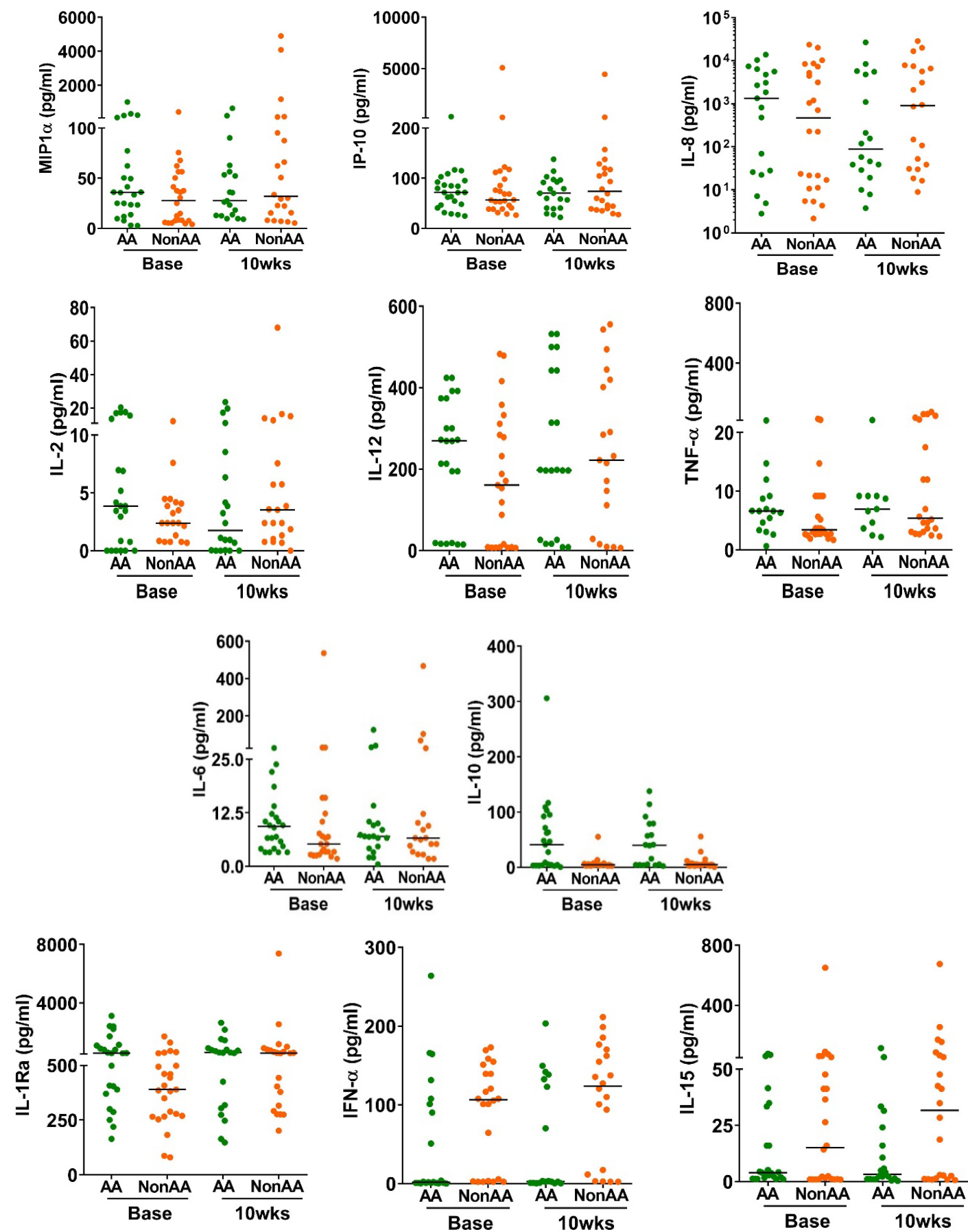

Supplement: Supplementary Figure S2 — Cytokine and chemokine responses in AA (n=29) and non-AA(n=28). [file crc-24-0112_supplementary_figure_s2_supps2.pdf]
