# Supplementary material for: Race-Related Differences in Sipuleucel-T Response among Men with Metastatic Castrate–Resistant Prostate Cancer
Source: Cancer Res Commun. 2024 Jun 10;4(7):1715–25. doi: 10.1158/2767-9764.CRC-24-0112 (PMC11240276; doi:10.1158/2767-9764.CRC-24-0112)
Supplement: Supplementary Figure S3B — Waterfall plot showing PSA change from baseline to week 10. [file crc-24-0112_supplementary_figure_s3b_supps3b.pdf]

Supplementary Figure S3B

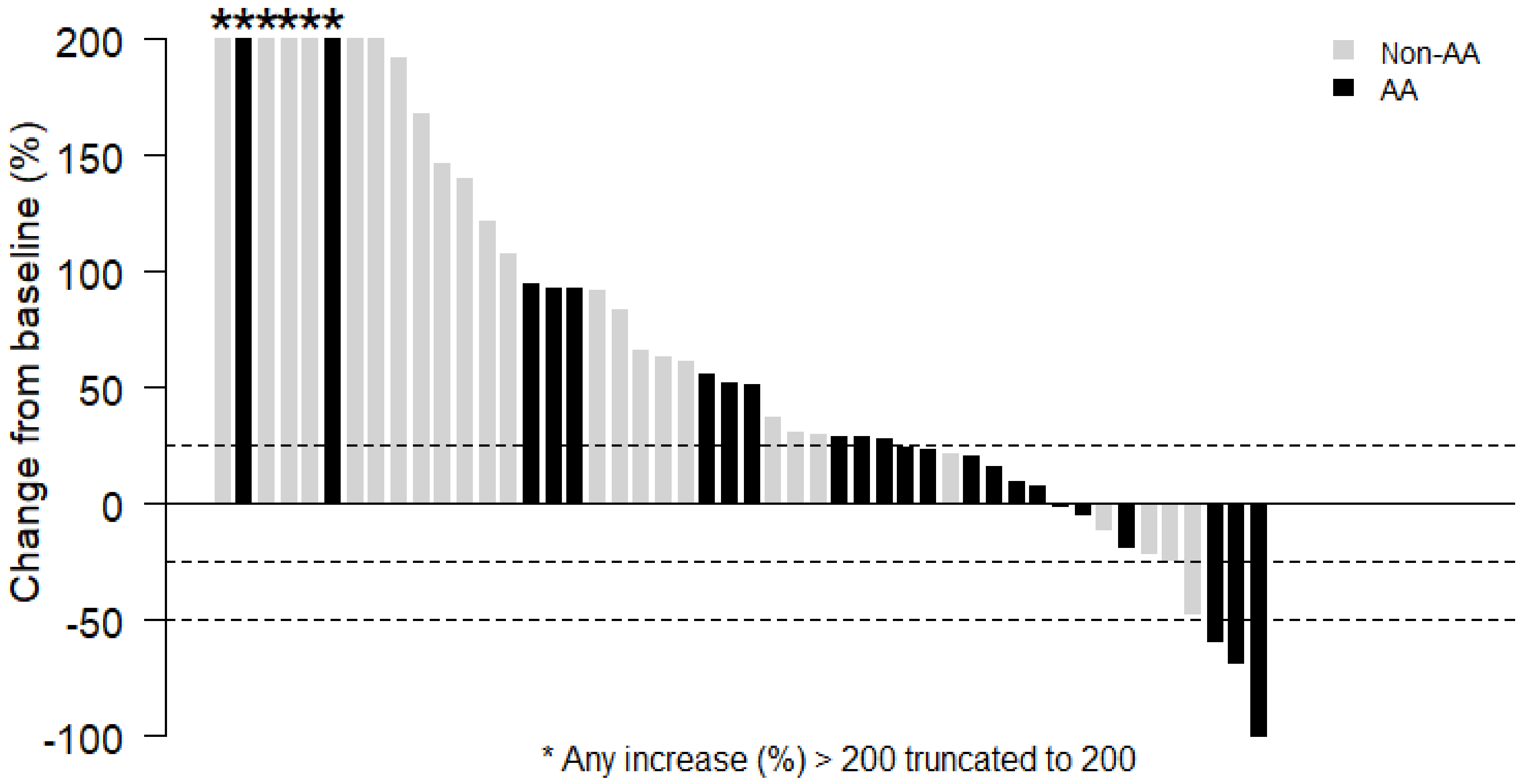

Supplementary Figure S3B. Waterfall plot showing PSA change from baseline to week 10.
