# Supplementary material for: Race-Related Differences in Sipuleucel-T Response among Men with Metastatic Castrate–Resistant Prostate Cancer
Source: Cancer Res Commun. 2024 Jun 10;4(7):1715–25. doi: 10.1158/2767-9764.CRC-24-0112 (PMC11240276; doi:10.1158/2767-9764.CRC-24-0112)
Supplement: Supplementary Figure S4 — Overall survival post-sipuleucel-T by race. [file crc-24-0112_supplementary_figure_s4_supps4.pdf]

Supplementary Figure S4

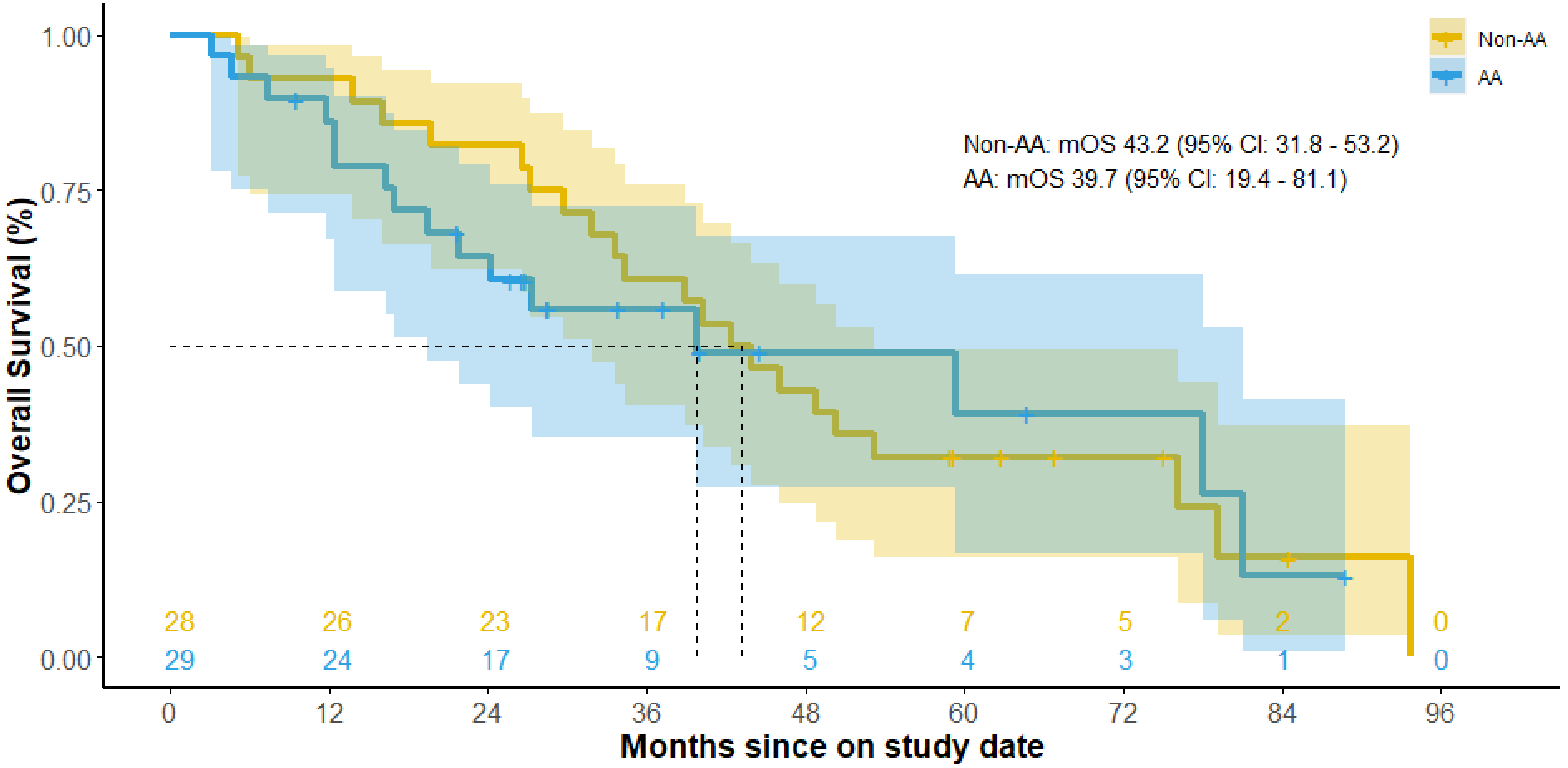

Supplementary Figure S4. Overall survival post-sipuleucel-T by race. Kaplan-Meier plot showing overall survival in in two racial groups. *AA* African Americans, *Non-AA* non-African Americans
