# Supplementary material for: Race-Related Differences in Sipuleucel-T Response among Men with Metastatic Castrate–Resistant Prostate Cancer
Source: Cancer Res Commun. 2024 Jun 10;4(7):1715–25. doi: 10.1158/2767-9764.CRC-24-0112 (PMC11240276; doi:10.1158/2767-9764.CRC-24-0112)
Supplement: Supplementary Table S1 — Antibody and IFN-γ responses and co-stimulatory and co-inhibitory marker expressions in mCRPC patients. [file crc-24-0112_supplementary_table_s1_suppst1.pdf]

Supplementary Table S1. Antibody and IFN-γ responses and co-stimulatory and co-inhibitory marker expressions in mCRPC patients

		African Americans	Non-African Americans		African Americans	Non-African Americans	
		**Baseline**	**Baseline**	**p value**	**10 weeks post sipuleucel-T**	**10 weeks post sipuleucel-T**	**p value**
**[a]IgM**	PA2024	1000 (50-100000)	1250 (100-4000000)	0.36	100000 (1000-8000000)	1000000(1000-10000000)	0.61
	PAP	1000 (1000-100000)	2500 (50-1000000)	0.9	100000 (10000-1000000)	100000 (1000-5000000)	0.5
	PSA	1000 (100-40000)	1000 (50-40000)	0.19	5500 (100-160000)	10000 (50-160000)	0.8
	PSMA	1000 (100-1000000)	20000 (50-100000)	0.6	5500 (50-160000)	40000 (10-1000000)	0.25
**[b]IFN-γ**	PA2024	8.165 (0-138.7)	7.33 (0-251.3)	0.83	16.25 (0.33-292.7)	14.17 (0-380.7)	0.77
	PAP	6 (0-112)	5.33 (0-241)	0.65	9.1 (0-225)	6.7 (0-218)	0.55
	PSA	5.5 (0-110)	5.3 (0-212.3)	0.94	4.8 (0-148.7)	7.1 (0-210.3)	0.64
	PSMA	10 (0-90)	9.6 (0-245.3)	0.73	9.5 (0-164)	12.5 (0-214.3)	0.48
**CD4 (%)**	CD4	64.4 (8.6-80.2)	67.85 (38.8-82.2)	0.24	62.8 (11.5-76.2)	62.65 (33-89.7)	0.61
	OX40	1.9 (0-30.7)	1.0 (0-46.1)	0.06	2.3 (0-49.6)	1.2 (0-33.1)	0.02
	ICOS	21.7 (0-69.5)	0.005 (0-0.7)	**<0.0007**	28.3 (0-70.2)	0.05 (0-2.2)	**<0.003**
	BTLA	44.1 (0.07-73.9)	4.4 (0-31.3)	**<0.0001**	42.9 (0.28-73)	5.5 (0-75)	0.16
	PD1	1.1 (0.3-12.7)	2.7 (0.02-9.1)	0.23	1.2 (0-14.6)	4.3 (0.4-18)	0.09
	CTLA-4	0.75 (0-34.2)	0.03 (0-44.3)	0.21	0.7 (0-29.5)	0.9 (0-57.2)	0.83
**CD8 (%)**	CD8	16.4 (4.8-44)	27 (4.8-68.5)	**<0.002**	15.9 (1.4-43)	26.1 (8.2-67.2)	0.02
	OX40	0.30 (0-7.5)	0 (0-20.6)	0.08	0.36 (0-15.8)	0 (0-25.8)	0.46
	ICOS	0.39 (0-21.5)	0 (0-0.47)	**<0.003**	0.68 (0-7.9)	0.03 (0-0.9)	**<0.003**
	BTLA	1.5 (0-31)	2.5 (0-38.6)	0.83	2.1 (0.13-45.3)	11 (0.26-63.7)	0.01
	PD1	1.07 (0-25.2)	3.33 (0.08-14.7)	0.04	1.5 (0-23.2)	5.5 (0.3-20)	0.01
	CTLA-4	0.55 (0-26.7)	0 (0-33.6)	0.28	1.0 (0-52)	1.5 (0-57.2)	0.95

The median and range values are shown. P-values <0.0032 are highlighted in bold indicating significantly higher expression in African Americans (*n*=29) vs non-African Americans (*n*=28).

[a]IgM response (values are above background at indicated dilution)

[b]IFN-γ producing T cells in $3x10^5$ peripheral blood mononuclear cells
