# Supplementary material for: Race-Related Differences in Sipuleucel-T Response among Men with Metastatic Castrate–Resistant Prostate Cancer
Source: Cancer Res Commun. 2024 Jun 10;4(7):1715–25. doi: 10.1158/2767-9764.CRC-24-0112 (PMC11240276; doi:10.1158/2767-9764.CRC-24-0112)
Supplement: Supplementary Table S2 — Th1 and Th2 cytokine and chemokine responses in mCRPC patients. [file crc-24-0112_supplementary_table_s2_suppst2.pdf]

Supplementary Table S2. Th_1 and Th_2 cytokine and chemokine responses in mCRPC patients

		African Americans	Non-African Americans	*P* value	African Americans	Non-African Americans	*P* value
		**Baseline**	**Baseline**		**10 weeks post sipuleucel-T**	**10 weeks post sipuleucel-T**	
**Cytokines (pg/mL)**	IL-2	3.86 (0-20.4)	2.4 (0.7-12.2)	0.41	1.755 (0-23.6)	3.53 (0-68)	0.29
	IL-12	269 (14.7-424)	160.4 (6-483)	0.14	197.3 (8-532)	221.8 (6-555)	0.9
	IFN-α	2.05 (.13-264)	106.6 (2.4-173)	0.006	2.86 (0.36-203)	123.9 (2.4-211.5)	0.01
	IFN-γ	6.45 (0.45-23.2)	8.12 (0.36-24)	0.94	3.45 (3.3-26.5)	3.28 (0.36-20.6)	0.4
	TNF-α	6.61 (0.63-20.3)	3.41 (1.7-31)	0.14	6.95 (2.2-23)	5.415 (2.3-77)	0.004
	GM-CSF	3.755 (0.2-55)	0.52 (0.2-2.3)	**<0.001**	2.98 (0.32-32.7)	0.59 (0.2-14.3)	0.02
	IL-15	3.95 (1.1-75.6)	15.17 (0.86-650)	0.93	3.2 (0.3-113.5)	31.6 (0.66-675.3)	0.07
	IL-4	0.605 (0.06-31.4)	1.16 (0.53-26.4)	0.02	0.53 (0.06-44.1)	11.05 (0.6-44.1)	0.01
	IL-6	9.3 (3.2-641.6)	5.18 (1.7-536)	0.13	6.9 (0.46-125)	6.6 (1.76-468)	0.81
	IL-10	40.84 (0.25-306)	4.19 (2.2-55)	0.009	39.9 (2.2-138)	4.9 (0.25-55.6)	0.005
**Chemokines (pg/mL)**	IP-10	71.6 (24.6-306)	57.08 (26.4-5080)	0.72	70.3 (22.1-137.7)	73.88 (27.5-4443)	0.43
	MIP-1α	35.9 (2.7-1023)	27.7 (4.1-435)	0.26	27.6 (9.2-650)	32 (5.3-4904)	0.72
	CCL4	216.7 (53-651)	87.32 (18.2-1143)	**<0.0003**	201.4 (67.6-371)	167.2 (67.7-1908)	0.7
	CCL5	56152 (3917-123107)	12304 (1044-54264)	**<0.0001**	63406 (7861-123750)	11724 (514-69436)	**<0.0007**
	MCP1	465.4 (45.5-3688)	624.6 (81.5-4405)	0.51	402.4 (604-3733)	1448 (70.7-4524)	0.03
	IL-8	1346 (2.8-13890)	471.9 (2.1-23745)	0.65	88.61 (3.75-26928)	907.1 (8.9-28911)	0.3

The median and range values are shown. P-values <0.0032 are highlighted in bold indicating significantly higher expression in African Americans (*n*=29) vs non-African Americans (*n*=28).
