# Supplementary material for: Race-Related Differences in Sipuleucel-T Response among Men with Metastatic Castrate–Resistant Prostate Cancer
Source: Cancer Res Commun. 2024 Jun 10;4(7):1715–25. doi: 10.1158/2767-9764.CRC-24-0112 (PMC11240276; doi:10.1158/2767-9764.CRC-24-0112)
Supplement: Supplementary Table S3 — Summary of significant responses before and after sipuleucel-T in mCRPC patients. [file crc-24-0112_supplementary_table_s3_suppst3.pdf]

Supplementary Table S3. Summary of significant responses before and after sipuleucel-T in mCRPC patients

		African Americans	Non-African Americans	p value	African Americans	Non-African Americans	p value
		**Baseline**	**Baseline**		**10 weeks post sipuleucel-T**	**10 weeks post sipuleucel-T**	
**CD4/CD8**	CD4-ICOS	21.7 (0-69.5)	0.005 (0-0.7)	**0.0007**	28.3 (0-70.2)	0.05 (0-2.2)	**0.003**
	CD4-BTLA	44.1 (0.07-73.9)	4.4 (0-31.3)	**0.0001**	42.9 (0.28-73)	5.5 (0-75)	0.16
	CD8-ICOS	0.39 (0-21.5)	0 (0-0.47)	**0.003**	0.68 (0-7.9)	0.03 (0-0.9)	**0.003**
**Cytokine/ chemokines**	GM-CSF	3.755 (0.2-55)	0.52 (0.2-2.3)	**0.001**	2.98 (0.32-32.7)	0.59 (0.2-14.3)	0.02
	CCL4	216.7 (53-651)	87.32 (18.2-1143)	**0.0003**	201.4 (67.6-371)	167.2 (67.7-1908)	0.7
	CCL5	56152 (3917-123107)	12304 (1044-54264)	**0.0001**	63406 (7861-123750)	11724 (514-69436)	**0.0007**

The median and range values are shown. P-values <0.0032 are highlighted in bold indicating significantly higher expression in African Americans (*n*=29) vs non-African Americans (*n*=28).
